# Anæsthesia Produced by Inhalations of Chloroform, and Kept up by Subcutaneous Injection of Morphia

**Published:** 1865-04

**Authors:** 


ANÆSTHESIA PRODUCED BY INHALATIONS OF CHLOROFORM, AND KEPT UP BY SUBCUTANEOUS INJECTION OF MORPHIA.—Experiments have lately been made upon dogs, by a committee appointed by the Medical Society of Versailles, to test the value of the above method. Five experiments were undertaken. On the first dog chloroform inhalation alone caused anæsthesia for nineteen minutes; with inhalation and the injection of about half a grain of morphine, thirty-six minutes. On a second dog, with chloroform inhalation alone, the anæsthesia lasted thirty minutes; but the same inhalation followed by an injection of one grain of morphine caused an anæsthetic state of one hour and twenty-seven minutes. The same experiment being repeated, with an injection of one grain and one-tenth of morphine, produced anæsthesia for five hours and forty-four minutes. The committee state in their report that the prolongation of anæsthesia by subcutaneous injections of morphine must be admitted as a fact, although they do not wish to be over-positive, seeing that their experiments are but few, and, in one instance, performed twice upon the same animal.

